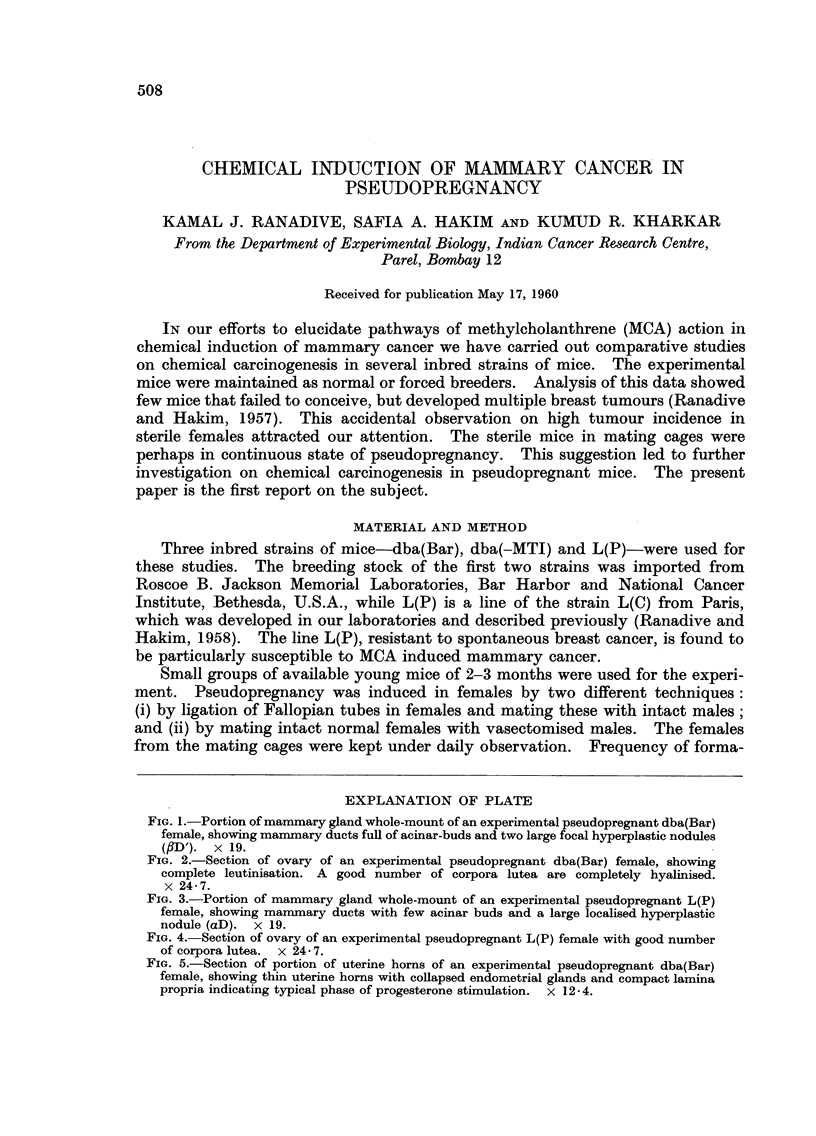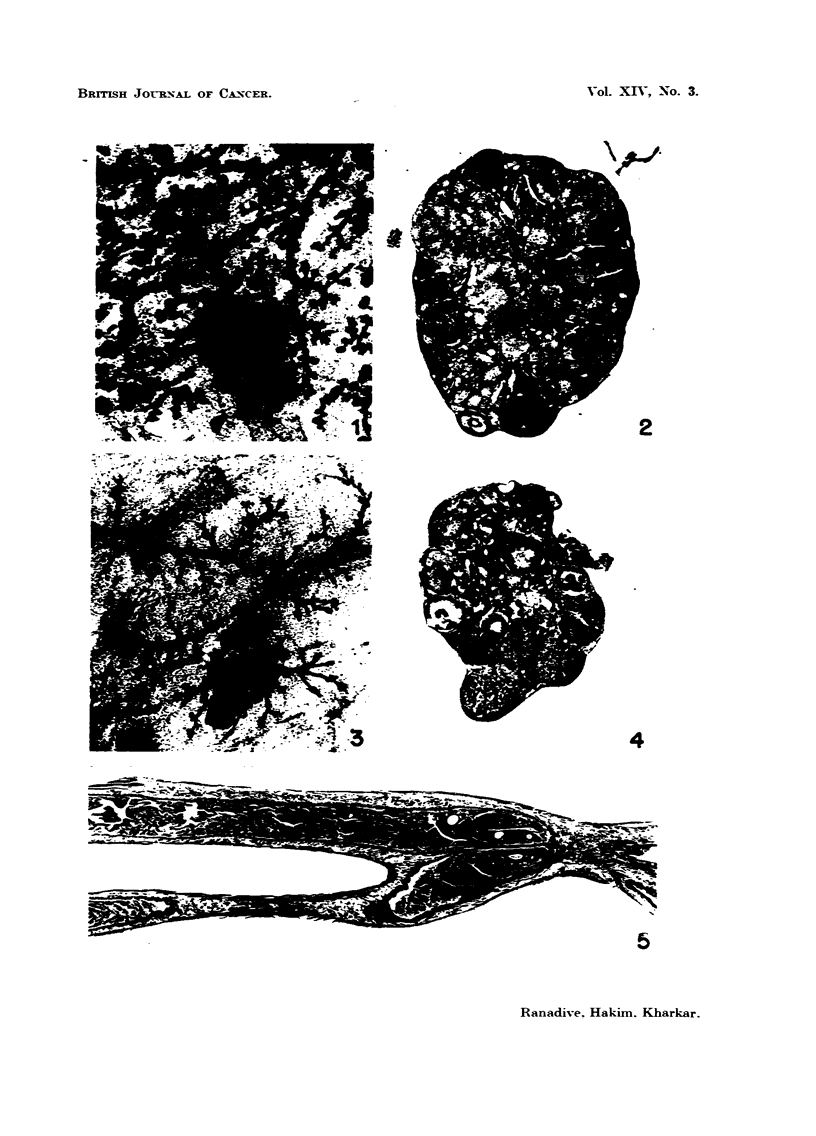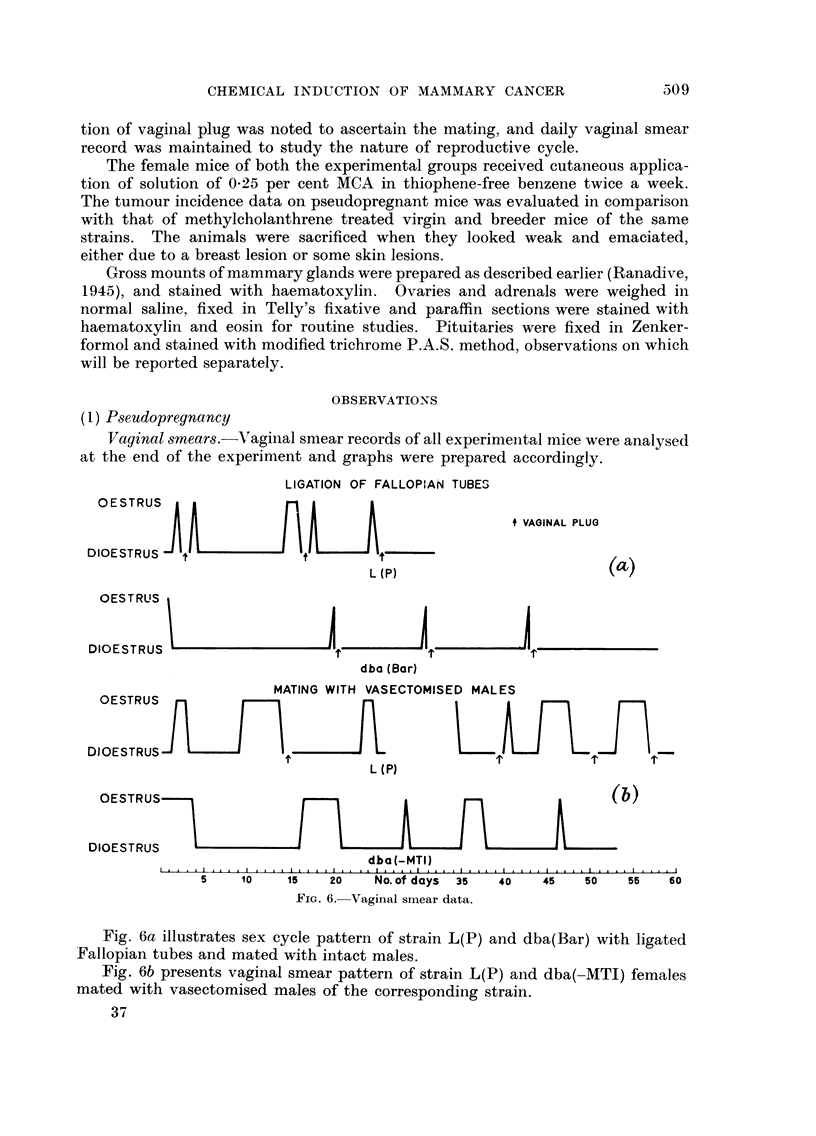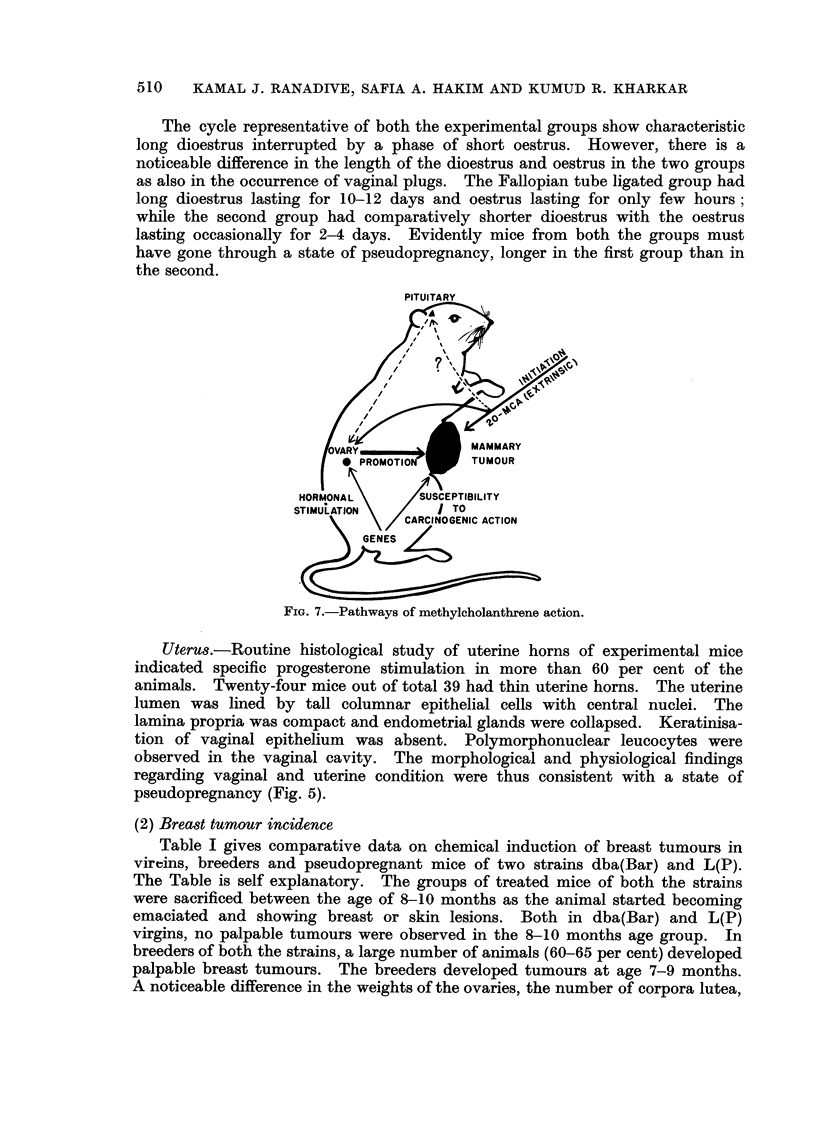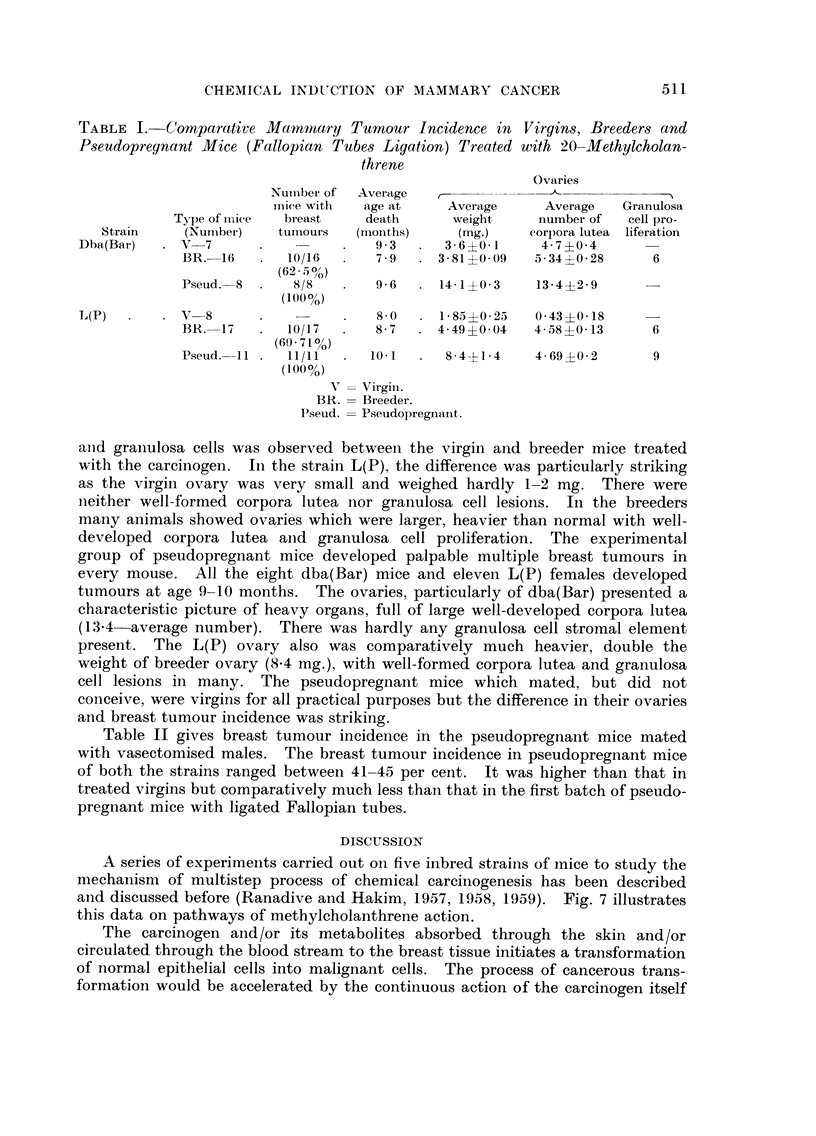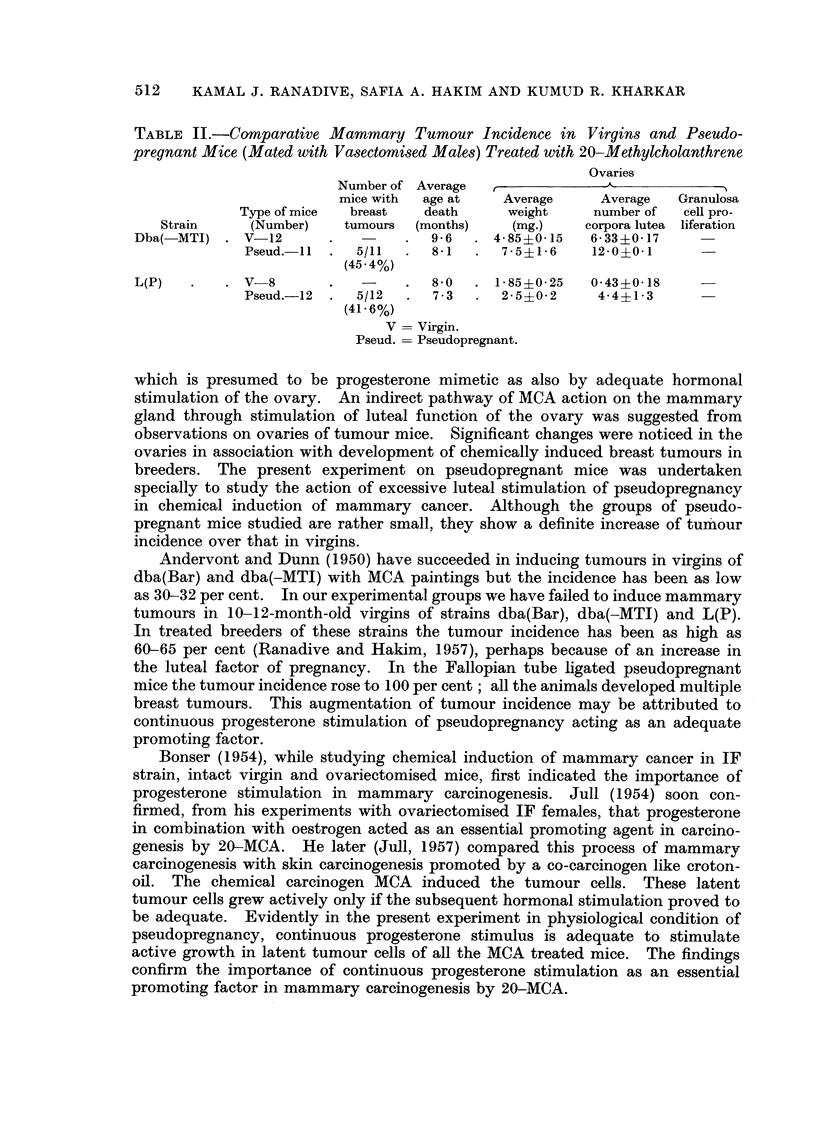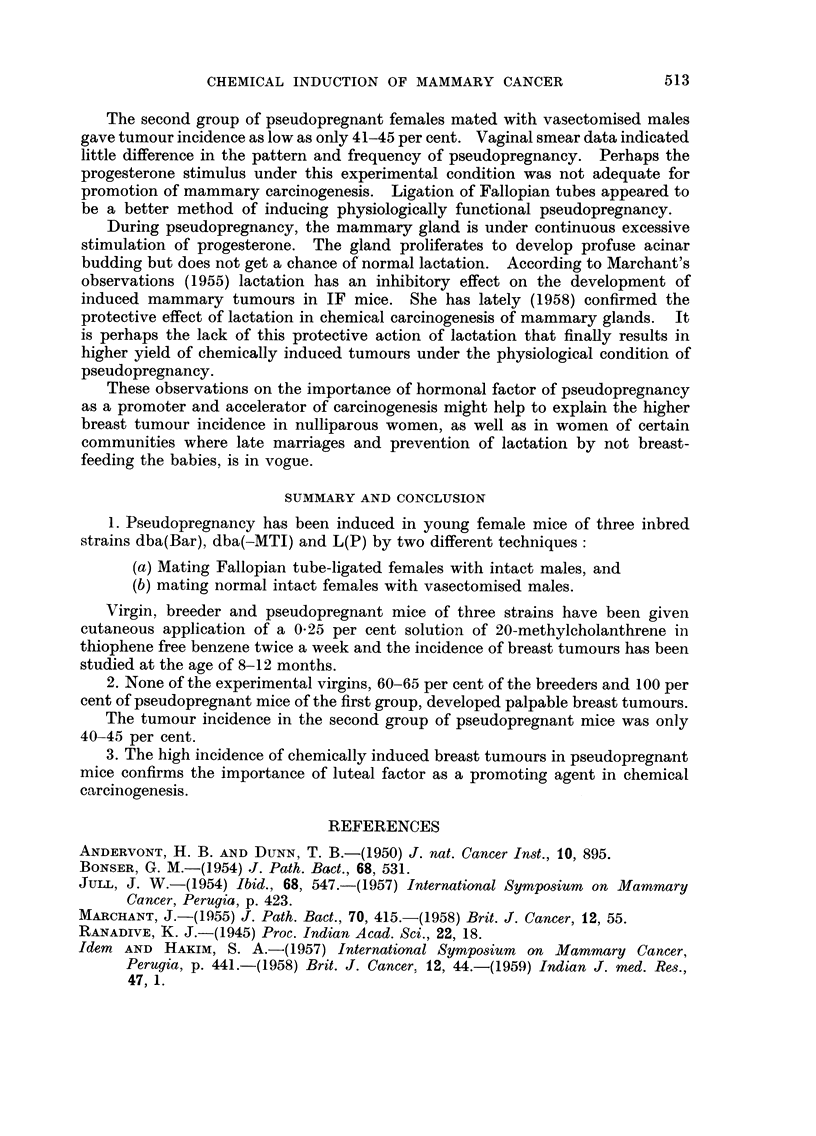# Chemical Induction of Mammary Cancer in Pseudopregnancy

**DOI:** 10.1038/bjc.1960.56

**Published:** 1960-09

**Authors:** Kamal J. Ranadiva, Safia A. Hakim, Kumud R. Kharkar

## Abstract

**Images:**


### CHEMICAL INDUCTION OF MAMMARY CANCER IN PSEUDOPREGNANCY

KAMAL J. RANADIVE, SAFIA A. HAKIM AND KUMUD R. KHARKAR

*From the Department of Experimental Biology, Indian Cancer Research Centre, Parel, Bombay* 12

Received for publication May 17, 1960

IN our efforts to elucidate pathways of methylcholanthrene (MCA) action in chemical induction of mammary cancer we have carried out comparative studies on chemical carcinogenesis in several inbred strains of mice. The experimental mice were maintained as normal or forced breeders. Analysis of this data showed few mice that failed to conceive, but developed multiple breast tumours (Ranadive and Hakim, 1957). This accidental observation on high tumour incidence in sterile females attracted our attention. The sterile mice in mating cages were perhaps in continuous state of pseudopregnancy. This suggestion led to further investigation on chemical carcinogenesis in pseudopregnant mice. The present paper is the first report on the subject.

### MATERIAL AND METHOD

Three inbred strains of mice—dba(Bar), dba(–MTI) and L(P)—were used for these studies. The breeding stock of the first two strains was imported from Roscoe B. Jackson Memorial Laboratories, Bar Harbor and National Cancer Institute, Bethesda, U.S.A., while L(P) is a line of the strain L(C) from Paris, which was developed in our laboratories and described previously (Ranadive and Hakim, 1958). The line L(P), resistant to spontaneous breast cancer, is found to be particularly susceptible to MCA induced mammary cancer.

Small groups of available young mice of 2–3 months were used for the experiment. Pseudopregnancy was induced in females by two different techniques: (i) by ligation of Fallopian tubes in females and mating these with intact males; and (ii) by mating intact normal females with vasectomised males. The females from the mating cages were kept under daily observation. Frequency of forma-

---

EXPLANATION OF PLATE

FIG. 1.—Portion of mammary gland whole-mount of an experimental pseudopregnant dba(Bar) female, showing mammary ducts full of acinar-buds and two large focal hyperplastic nodules ($\beta$D′). × 19.

FIG. 2.—Section of ovary of an experimental pseudopregnant dba(Bar) female, showing complete leutinisation. A good number of corpora lutea are completely hyalinised. × 24·7.

FIG. 3.—Portion of mammary gland whole-mount of an experimental pseudopregnant L(P) female, showing mammary ducts with few acinar buds and a large localised hyperplastic nodule ($\alpha$D). × 19.

FIG. 4.—Section of ovary of an experimental pseudopregnant L(P) female with good number of corpora lutea. × 24·7.

FIG. 5.—Section of portion of uterine horns of an experimental pseudopregnant dba(Bar) female, showing thin uterine horns with collapsed endometrial glands and compact lamina propria indicating typical phase of progesterone stimulation. × 12·4.

Ranadive, Hakim, Kharkar.

tion of vaginal plug was noted to ascertain the mating, and daily vaginal smear record was maintained to study the nature of reproductive cycle.

The female mice of both the experimental groups received cutaneous application of solution of 0·25 per cent MCA in thiophene-free benzene twice a week. The tumour incidence data on pseudopregnant mice was evaluated in comparison with that of methylcholanthrene treated virgin and breeder mice of the same strains. The animals were sacrificed when they looked weak and emaciated, either due to a breast lesion or some skin lesions.

Gross mounts of mammary glands were prepared as described earlier (Ranadive, 1945), and stained with haematoxylin. Ovaries and adrenals were weighed in normal saline, fixed in Telly's fixative and paraffin sections were stained with haematoxylin and eosin for routine studies. Pituitaries were fixed in Zenker-formol and stained with modified trichrome P.A.S. method, observations on which will be reported separately.

### OBSERVATIONS

### (1) *Pseudopregnancy*

*Vaginal smears.*—Vaginal smear records of all experimental mice were analysed at the end of the experiment and graphs were prepared accordingly.

FIG. 6.—Vaginal smear data.

Fig. 6*a* illustrates sex cycle pattern of strain L(P) and dba(Bar) with ligated Fallopian tubes and mated with intact males.

Fig. 6*b* presents vaginal smear pattern of strain L(P) and dba(–MTI) females mated with vasectomised males of the corresponding strain.

The cycle representative of both the experimental groups show characteristic long dioestrus interrupted by a phase of short oestrus. However, there is a noticeable difference in the length of the dioestrus and oestrus in the two groups as also in the occurrence of vaginal plugs. The Fallopian tube ligated group had long dioestrus lasting for 10–12 days and oestrus lasting for only few hours; while the second group had comparatively shorter dioestrus with the oestrus lasting occasionally for 2–4 days. Evidently mice from both the groups must have gone through a state of pseudopregnancy, longer in the first group than in the second.

FIG. 7.—Pathways of methylcholanthrene action.

*Uterus.*—Routine histological study of uterine horns of experimental mice indicated specific progesterone stimulation in more than 60 per cent of the animals. Twenty-four mice out of total 39 had thin uterine horns. The uterine lumen was lined by tall columnar epithelial cells with central nuclei. The lamina propria was compact and endometrial glands were collapsed. Keratinisation of vaginal epithelium was absent. Polymorphonuclear leucocytes were observed in the vaginal cavity. The morphological and physiological findings regarding vaginal and uterine condition were thus consistent with a state of pseudopregnancy (Fig. 5).

(2) *Breast tumour incidence*

Table I gives comparative data on chemical induction of breast tumours in vireins, breeders and pseudopregnant mice of two strains dba(Bar) and L(P). The Table is self explanatory. The groups of treated mice of both the strains were sacrificed between the age of 8–10 months as the animal started becoming emaciated and showing breast or skin lesions. Both in dba(Bar) and L(P) virgins, no palpable tumours were observed in the 8–10 months age group. In breeders of both the strains, a large number of animals (60–65 per cent) developed palpable breast tumours. The breeders developed tumours at age 7–9 months. A noticeable difference in the weights of the ovaries, the number of corpora lutea,

TABLE I.—*Comparative Mammary Tumour Incidence in Virgins, Breeders and Pseudopregnant Mice (Fallopian Tubes Ligation) Treated with 20–Methylcholanthrene*

| Strain | Type of mice (Number) | Number of mice with breast tumours | Average age at death (months) | Ovaries: Average weight (mg.) | Ovaries: Average number of corpora lutea | Ovaries: Granulosa cell proliferation |
|---|---|---|---|---|---|---|
| Dba(Bar) | V—7 | — | 9·3 | 3·6±0·1 | 4·7±0·4 | — |
| | BR.—16 | 10/16 (62·5%) | 7·9 | 3·81±0·09 | 5·34±0·28 | 6 |
| | Pseud.—8 | 8/8 (100%) | 9·6 | 14·1±0·3 | 13·4±2·9 | — |
| L(P) | V—8 | — | 8·0 | 1·85±0·25 | 0·43±0·18 | — |
| | BR.—17 | 10/17 (60·71%) | 8·7 | 4·49±0·04 | 4·58±0·13 | 6 |
| | Pseud.—11 | 11/11 (100%) | 10·1 | 8·4±1·4 | 4·69±0·2 | 9 |

V = Virgin.

BR. = Breeder.

Pseud. = Pseudopregnant.

and granulosa cells was observed between the virgin and breeder mice treated with the carcinogen. In the strain L(P), the difference was particularly striking as the virgin ovary was very small and weighed hardly 1–2 mg. There were neither well-formed corpora lutea nor granulosa cell lesions. In the breeders many animals showed ovaries which were larger, heavier than normal with well-developed corpora lutea and granulosa cell proliferation. The experimental group of pseudopregnant mice developed palpable multiple breast tumours in every mouse. All the eight dba(Bar) mice and eleven L(P) females developed tumours at age 9–10 months. The ovaries, particularly of dba(Bar) presented a characteristic picture of heavy organs, full of large well-developed corpora lutea (13·4—average number). There was hardly any granulosa cell stromal element present. The L(P) ovary also was comparatively much heavier, double the weight of breeder ovary (8·4 mg.), with well-formed corpora lutea and granulosa cell lesions in many. The pseudopregnant mice which mated, but did not conceive, were virgins for all practical purposes but the difference in their ovaries and breast tumour incidence was striking.

Table II gives breast tumour incidence in the pseudopregnant mice mated with vasectomised males. The breast tumour incidence in pseudopregnant mice of both the strains ranged between 41–45 per cent. It was higher than that in treated virgins but comparatively much less than that in the first batch of pseudopregnant mice with ligated Fallopian tubes.

### DISCUSSION

A series of experiments carried out on five inbred strains of mice to study the mechanism of multistep process of chemical carcinogenesis has been described and discussed before (Ranadive and Hakim, 1957, 1958, 1959). Fig. 7 illustrates this data on pathways of methylcholanthrene action.

The carcinogen and/or its metabolites absorbed through the skin and/or circulated through the blood stream to the breast tissue initiates a transformation of normal epithelial cells into malignant cells. The process of cancerous transformation would be accelerated by the continuous action of the carcinogen itself

TABLE II.—*Comparative Mammary Tumour Incidence in Virgins and Pseudopregnant Mice (Mated with Vasectomised Males) Treated with 20–Methylcholanthrene*

| | | | | Ovaries | | |
|---|---|---|---|---|---|---|
| Strain | Type of mice (Number) | Number of mice with breast tumours | Average age at death (months) | Average weight (mg.) | Average number of corpora lutea | Granulosa cell proliferation |
| Dba(—MTI) | V—12 | — | 9·6 | 4·85±0·15 | 6·33±0·17 | — |
| | Pseud.—11 | 5/11 (45·4%) | 8·1 | 7·5±1·6 | 12·0±0·1 | — |
| L(P) | V—8 | — | 8·0 | 1·85±0·25 | 0·43±0·18 | — |
| | Pseud.—12 | 5/12 (41·6%) | 7·3 | 2·5±0·2 | 4·4±1·3 | — |

V = Virgin.
Pseud. = Pseudopregnant.

which is presumed to be progesterone mimetic as also by adequate hormonal stimulation of the ovary. An indirect pathway of MCA action on the mammary gland through stimulation of luteal function of the ovary was suggested from observations on ovaries of tumour mice. Significant changes were noticed in the ovaries in association with development of chemically induced breast tumours in breeders. The present experiment on pseudopregnant mice was undertaken specially to study the action of excessive luteal stimulation of pseudopregnancy in chemical induction of mammary cancer. Although the groups of pseudopregnant mice studied are rather small, they show a definite increase of tumour incidence over that in virgins.

Andervont and Dunn (1950) have succeeded in inducing tumours in virgins of dba(Bar) and dba(–MTI) with MCA paintings but the incidence has been as low as 30–32 per cent. In our experimental groups we have failed to induce mammary tumours in 10–12-month-old virgins of strains dba(Bar), dba(–MTI) and L(P). In treated breeders of these strains the tumour incidence has been as high as 60–65 per cent (Ranadive and Hakim, 1957), perhaps because of an increase in the luteal factor of pregnancy. In the Fallopian tube ligated pseudopregnant mice the tumour incidence rose to 100 per cent ; all the animals developed multiple breast tumours. This augmentation of tumour incidence may be attributed to continuous progesterone stimulation of pseudopregnancy acting as an adequate promoting factor.

Bonser (1954), while studying chemical induction of mammary cancer in IF strain, intact virgin and ovariectomised mice, first indicated the importance of progesterone stimulation in mammary carcinogenesis. Jull (1954) soon confirmed, from his experiments with ovariectomised IF females, that progesterone in combination with oestrogen acted as an essential promoting agent in carcinogenesis by 20–MCA. He later (Jull, 1957) compared this process of mammary carcinogenesis with skin carcinogenesis promoted by a co-carcinogen like croton-oil. The chemical carcinogen MCA induced the tumour cells. These latent tumour cells grew actively only if the subsequent hormonal stimulation proved to be adequate. Evidently in the present experiment in physiological condition of pseudopregnancy, continuous progesterone stimulus is adequate to stimulate active growth in latent tumour cells of all the MCA treated mice. The findings confirm the importance of continuous progesterone stimulation as an essential promoting factor in mammary carcinogenesis by 20–MCA.

The second group of pseudopregnant females mated with vasectomised males gave tumour incidence as low as only 41–45 per cent. Vaginal smear data indicated little difference in the pattern and frequency of pseudopregnancy. Perhaps the progesterone stimulus under this experimental condition was not adequate for promotion of mammary carcinogenesis. Ligation of Fallopian tubes appeared to be a better method of inducing physiologically functional pseudopregnancy.

During pseudopregnancy, the mammary gland is under continuous excessive stimulation of progesterone. The gland proliferates to develop profuse acinar budding but does not get a chance of normal lactation. According to Marchant's observations (1955) lactation has an inhibitory effect on the development of induced mammary tumours in IF mice. She has lately (1958) confirmed the protective effect of lactation in chemical carcinogenesis of mammary glands. It is perhaps the lack of this protective action of lactation that finally results in higher yield of chemically induced tumours under the physiological condition of pseudopregnancy.

These observations on the importance of hormonal factor of pseudopregnancy as a promoter and accelerator of carcinogenesis might help to explain the higher breast tumour incidence in nulliparous women, as well as in women of certain communities where late marriages and prevention of lactation by not breast-feeding the babies, is in vogue.

### SUMMARY AND CONCLUSION

1. Pseudopregnancy has been induced in young female mice of three inbred strains dba(Bar), dba(-MTI) and L(P) by two different techniques :

(*a*) Mating Fallopian tube-ligated females with intact males, and
(*b*) mating normal intact females with vasectomised males.

Virgin, breeder and pseudopregnant mice of three strains have been given cutaneous application of a 0·25 per cent solution of 20-methylcholanthrene in thiophene free benzene twice a week and the incidence of breast tumours has been studied at the age of 8–12 months.

2. None of the experimental virgins, 60–65 per cent of the breeders and 100 per cent of pseudopregnant mice of the first group, developed palpable breast tumours.

The tumour incidence in the second group of pseudopregnant mice was only 40–45 per cent.

3. The high incidence of chemically induced breast tumours in pseudopregnant mice confirms the importance of luteal factor as a promoting agent in chemical carcinogenesis.

### REFERENCES

Andervont, H. B. and Dunn, T. B.—(1950) *J. nat. Cancer Inst.*, **10**, 895.

Bonser, G. M.—(1954) *J. Path. Bact.*, **68**, 531.

Jull, J. W.—(1954) *Ibid.*, **68**, 547.—(1957) *International Symposium on Mammary Cancer, Perugia*, p. 423.

Marchant, J.—(1955) *J. Path. Bact.*, **70**, 415.—(1958) *Brit. J. Cancer*, **12**, 55.

Ranadive, K. J.—(1945) *Proc. Indian Acad. Sci.*, **22**, 18.

*Idem* and Hakim, S. A.—(1957) *International Symposium on Mammary Cancer, Perugia*, p. 441.—(1958) *Brit. J. Cancer*, **12**, 44.—(1959) *Indian J. med. Res.*, **47**, 1.